# Propofol sedation safety concerns in pediatric endoscopy: Special considerations in the Japanese medical environment

**DOI:** 10.1002/deo2.70000

**Published:** 2024-09-30

**Authors:** Daisuke Murakami, Masayuki Yamato

**Affiliations:** ^1^ Department of Gastroenterology Tokyo Women's Medical University Yachiyo Medical Center Chiba Japan; ^2^ Institute of Advanced Biomedical Engineering and Science Tokyo Women's Medical University Tokyo Japan

To the Editor,

Kudo and colleagues^1^ clearly demonstrated that intravenous propofol anesthesia significantly shortened procedure time in pediatric endoscopy, with no adverse events observed. Subsequent to the revision of Japanese guidelines for endoscopic sedation,^2^ gradual adoption of propofol sedation in adult endoscopic procedures is noted. Importantly, however, this guideline explicitly excludes pediatric populations. Additionally, this guideline refrains from explicitly stating whether non‐anesthesiologists can safely administer propofol sedation in endoscopy units. Gastroenterologists should understand that propofol is not recommended for anesthetic induction below the age of 3 years or for anesthetic maintenance below 2 months of age. As discussed in this paper, propofol is contraindicated for pediatric intensive care unit sedation due to the risk of propofol infusion syndrome; thus, excessive use should be avoided. Actually, our hospital witnessed an accidental intensive care unit death of a pediatric patient sedated with propofol.

Our concern stems from the fact that current evidence regarding safe propofol sedation for endoscopy is based predominantly on international reports. The Japanese medical environment differs significantly from those abroad. While anesthesiologists in many other countries routinely administer sedation, the current structure and resource allocation in Japan makes it practically infeasible to have anesthesiologists permanently stationed in endoscopy units. Unlike benzodiazepines, propofol has a narrow pharmacological range between states of sedation with maintained respiration and that of general anesthesia where spontaneous breathing ceases, making it prone to easily induce over‐sedation. Therefore, the guideline also emphasizes the use of various devices supporting safe propofol administration; consequently, continuous administration via a target‐controlled infusion pump is recommended, and the importance of capnography and/or electroencephalography is stressed (in Japan, widespread adoption of this monitoring in pediatric patients is insufficient). The methodology in Kudo et al.^1^ did not address these recommendations.

Given the aforementioned precautions for propofol use in pediatric patients, endoscopic sedation in children using propofol warrants more careful consideration, prioritizing safety.

## CONFLICT OF INTEREST STATEMENT

None.

## ETHICS STATEMENT

‐ Approval of the research protocol by an Institutional Reviewer Board: N/A.

‐ Informed consent: N/A.

‐ Registry and the Registration No. of the study/trial: N/A.

‐ Animal studies: N/A.
